# Women’s health status in urban Ghana: dimensions and differentials using short form 36

**DOI:** 10.1186/s12955-018-0894-y

**Published:** 2018-04-24

**Authors:** Faustina Frempong-Ainguah, Claire E. Bailey, Allan G. Hill

**Affiliations:** 1 Regional Institute for Population Studies, College of Humanities, University of Ghana, P. O. Box LG 96, Legon, Accra, Ghana; 2 Department of Social Statistics and Demography, Faculty of Social and Human Sciences, University of Southampton, Highfield, Southampton, UK

**Keywords:** Women’s health, Socio-demographic differentials, Short Form-36 eight scales

## Abstract

**Background:**

Global discourse on population, health and development have placed women’s health issues at the top of development agenda. Women’s reproductive health has received some attention in Ghana since the mid-1990s. However, studies on women’s general health status, dimensions and the differentials in a rapidly growing urban setting is poorly understood and under-researched.

This study sought to examine the various pathways in which individual socio-demographic factors, economic characteristics and endowment influence self-assessed health status among women living in the city of Accra, Ghana.

**Methods:**

The paper draws on a cross-sectional study carried out in 2008 and 2009 using a representative sample of urban women 20 years and older (*n* = 2814). Multivariate stepwise linear regression models were performed to investigate the influence of socio-demographic, economic and health indicators on health-related quality of life, measured by eight sub-scales of the Short Form-36 (SF-36). Interaction effects between some demographic and socio-economic variables were also performed.

**Results:**

The analyses show diverse relationships between demographic, socio-economic and health indicators and health outcomes assessed using eight SF-36 sub-scales. Education, disease symptoms and age of the respondent were the most significant factors influencing good overall health status. Interestingly, age has no significant effect on mental health after controlling for all other explanatory variables.

**Conclusions:**

The findings show that health issues are multi-faceted requiring socio-cultural, health and economic policy interventions. Investing in women’s education is important to improve health status. There is also the need for more effective collaboration across various sectors to improve the health and well-being of women in general. Ageing has increasing relationship with poor physical health status and the elderly should be given needed attention and support.

## Background

Development targets, such as the Millennium Development Goals and the Sustainable Development Goals, have repeatedly emphasized health as a key aspect of development in need of attention. Measuring a population’s health status is important as it helps to identify major health problems confronting any group of people within a community. It also helps to set policy goals to improve the provision of health services and to monitor the effectiveness of medical interventions. Global discourse on population, health and development have placed women’s health issues at the top of development agenda. Women’s health has received some attention in Ghana in reproductive health since the mid-1990s. However, studies on women’s general health status or intra-urban quality of life health differentials have not received much attention in the past, nor the present. This paper adopts Short Form-36 (SF-36) self-assessed quality of life measures to assess health status among women 20 years and older living in the city of Accra, Ghana. Examining their general health status would be of vital importance to policy making.

Measuring individual’s health status or well-being is complex and challenging especially in developing countries, including Ghana [1, 2]. Estimates of expectation of life and mortality summaries are often used as proxy indicators at the population level [3, 4]. However, these aggregate measures only refer to the length of people’s lives, rather than the quality of life of those alive [5]. Hence are not sensitive indicators of a nation’s health status [6]. World Health Organisation (WHO) defines health broadly as not only the absence of disease and infirmity but also having complete physical, mental and social wellbeing [7]. These different dimensions of health status help to assess a population’s health in a meaningful and reliable way [8, 9]. It is worth noting that the changing roles of women present concern for their health in the broader health and wellbeing discourse. The level, dimensions of health status and the extent of intra-urban health differentials among women in a rapidly growing city of Accra and their relations to overall health status are poorly understood, and are under-researched.

Healthcare in Ghana is provided mainly by the government through the Ghana Health Service [10, 11], and it is organised around curative primary health care services [12–14]. Treating infectious diseases is very important in Ghana; however, the tendency to emphasise curative medicine at the expense of public health has serious repercussions [14]. Urban health in Ghana, as in other sub-Saharan African countries, received little attention until barely three decades ago [15, 16]. Research by international donors and local health sector activities has focused attention on primary health care and some tropical diseases to the neglect of others [12, 17, 18]. This has influenced the development strategies of governments and health-related agencies in Ghana. Although these policies have led to a reduction in mortality rates and some tropical diseases [19], large gaps remain concerning the level and extent of women’s health problems that are not tropical diseases [20, 21]. Thus, it is possible to live longer but be in a deteriorating health state [1, 3]. Policy makers and health care providers cannot reduce women’s ill health by simply extending policies that have been effective in improving child health. Thus, equal attention must be devoted to women’s health problems in order to understand their morbidity patterns and health status. A thorough assessment of health status must go beyond a disease-specific approach to have a full perspective of the population’s health in general [9, 22].

Accra, like most cities in sub-Saharan Africa, has been experiencing rapid urbanisation, with an average annual growth rate exceeding 5 % per annum between 1984 and 2000, and a little over 2 % per annum between 2000 and 2010 [23, 24]. Living in an urban area offers many opportunities, including better healthcare services, good education, employment opportunities, access to information and social amenities thereby increasing living standards [25, 26]. Although these opportunities exist, they might not be uniformly distributed. Rapid and unplanned urban growth, with unmatched socio-economic development, often place the health of the people at risk [15, 27, 28]. Despite the rapid urbanisation taking place in Accra, there have been few detailed case studies to examine the morbidity patterns and intra-urban health differentials within the city of Accra [29–31]. Therefore, disaggregated intra-urban health data is most useful for understanding their health patterns and differentials. Hence, a study that examines the relationships among the demographic, socio-economic and disease symptoms that influence the health status of women is appropriate. This will help health planners to ascertain the true health status of women, as well as identify their healthcare needs outside their reproductive challenges.

Women are faced with certain social constraints, including limited education, reproductive rights and household roles, which limit their ability to secure stable employment [15, 32]. This makes them less economically self-sufficient than men. Women’s assigned social roles can be very stressful, especially when combining household chores, reproductive roles, as primary caregivers to their immediate household, and the extended family, as well as making a living [31–33]. These exert much pressure on their physical and emotional wellbeing, which invariably affects their overall health status [34–36]. Good health status among women improves not only their own wellbeing, but also impact on the health of their children and other family members [37, 38].

Self-assessed measures of health status broadly consist of all factors which directly or indirectly tap into various facets of life issues. The use of self-assessed quality of life measures has been described as one of the most important client-oriented health outcomes [39–41]. It has been recommended by WHO for monitoring health as a tool for disease risk screening [42–44] and as standard part of clinical care [45]. These diverse perspectives will offer a better understanding of the intra-urban health patterns and provide a broad snapshot of some aspects of health among the study population as opposed to aggregated urban information portraying the unit as a homogenous group. Different social or age groups might be healthy or unhealthy in different ways depending on how they assess their health. Similarly, different health-related behaviours, material circumstances, socio-cultural and environmental factors may affect an individual’s measurement of health. This paper examines the socio-demographic and economic factors on general health or well-being and the plausible ways by which differentials may exist. Little research has been conducted into the study of health patterns and differentials using this approach and, relatively little research has been done looking at all eight health sub-scales of the SF-36. Understanding the key components of health can help inform policies and interventions to improve the different aspects of health and health outcomes in the general population.

## Methods

### Data and study population

The study draws on a representative sample of 2809 women 20 years and older from the second wave of the Women’s Health Study of Accra (WHSA). The 2008/9 round of WHSA is a follow up of the wave I conducted in 2003. It is a representative cross-sectional study of the city of Accra. It was designed as a community-based assessment of the health conditions observed in the general population of women aged 18 years and older living in private households. The study employed a multi-stage sample design from the Ghana Statistical Service. The study area consisted of 1731 Enumeration Areas (EAs) or primary sampling units (PSU), with a population between 1.7 million and 2.3 million living in 363,540 households between March 2000 and October 2010 [23, 24]. At the first stage of sampling, 200 EAs were selected and listing exercise of all eligible households in the selected EAs was carried out. The list of names and detailed addresses of individuals within the canvassed households in the selected EAs formed the frame for the selection of individuals. At the second stage of selection, 16 individuals were systematically selected, one per household, from the detailed list of women. The list yielded 3068 respondents and complete interview were obtained from 2814. Of these, 2809 had complete information on the eight SF-36 sub-scales. The study was conducted by the Institute of Statistical, Social and Economic Research, University of Ghana and the Department of Global Health and Population, Harvard School of Public Health. Interviews were conducted using structured questionnaires, after respondents had given their consent. The protocol comprised a household roster and demographic module, an individual interview for socio-economic information, the SF-36 health questions, self-reported illnesses, health care utilisation, medical history and lifestyle patterns, among others.

The survey instrument was translated from English to the local language and piloted to test its suitability among the study population. Experienced field personnel were recruited, trained and used to gather the information between October 2008 and June 2009 [46].

The SF-36 is a standardised, multi-purpose, multi-item scale used to assess the health of the general population or patients across eight-health concepts. These include physical functioning (PF), role limitation due to physical health problems (RP), bodily pain (BP), role limitations due to personal or emotional problems (RE), general health perception (GH), social functioning (SF), vitality (VT), and general mental health (MH). In addition, the eight sub-scales yield two summary scores (Physical Component Summary- (PCS) and Mental Component Summary (MCS)) relating to physical functioning and emotional wellbeing [47]. This paper draws on the notion that there are various pathways through which individual characteristics, self-endowed traits and lifestyles may influence overall health outcomes. The paper seeks to understand the general characteristics and levels of poor health across the study population using the eight health sub-scales, and to examine how well do the selected demographic, socio-economic and health-related characteristics contribute to understanding relative poor health by each of the eight health sub-scales.

### Variables

The outcome variables for the analysis are the eight health sub-scales derived from the SF-36. The eight sub-scales are: physical functioning, role limitation due to physical health, bodily pain, role limitations due to emotional problems, general health perception, social functioning, vitality or fatigue, and general mental health. These eight sub-scales aggregate between 2 and 10 items each and are scored 0-100, using the RAND 36-Item Health Survey version 1.0 scoring procedure [48]. This method of estimation was used instead of the normed based to make it comparable to earlier studies conducted in the WHSA wave 1 and also because the focus was on using the item-level data to obtain the eight sub-scales. A higher value on any of these eight sub-scales depict a favourable or good self-perceived health status while lower scores indicate poorer health status*.*

### Explanatory variables

The explanatory variables have been grouped into demographic, socio-economic variables, health behaviours and measures of disease. The explanatory variables were selected based on the literature and the analytical framework for this study. The explanatory variables selected included age, marital status, parity, ethnicity and social networking, while the socio-economic variables focused on educational level and wealth status of household measured using household assets and using principal component analysis to create quintiles. Health behaviour variables comprised body mass index (BMI) and disease symptoms experienced one month prior to the study. Age measured in completed years was classified as a categorical indicator into five groupings: 20-29, 30-39, 40-49, 50-59, 60+. Education was categorised into: no formal schooling, primary or lower, junior high school (JHS) and secondary or higher (Sec+). Household assets were used as a proxy indicator to compute the wealth status of the household in which the woman lives using principal component analysis. The first factor loading produced was used to create quintiles: ‘1’ = Poorest, ‘2’ = Poorer, ‘3’ = Middle, ‘4’ = Richer’, ‘5′ = Richest. Marital status was classified as currently in union, formerly in union and never married. Ethnicity was classified into four major groups- Akan, Ewe, Ga-Dangme and other ethnic groups. Social network was a derived dummy variable classified into good (code = 0) or poor networking (code =1) from questions asked to the respondent about her personal relationship with others, community participation, dealing with conflict and tension in the past 30 days prior to the survey. Disease symptom was a derived variable from 14 conditions: chest pains or discomfort at rest or with exertion, palpitations, shortness of breath with exertion or when lying flat, wheezing at rest or with exertion, tightness in chest, pain in legs while walking, swollen legs and dizzy spells. Respondents who indicated ‘no’ to all these was coded as 0 = no disease symptom, yes to anyone of the disease symptoms was coded ‘1= single symptom’ and code 2 yes to any two or more disease symptoms classified as multiple symptom experienced.

### Analytic technique

Univariate analysis was first performed to produce summary statistics for each of the outcome variables, and frequency distributions were generated for the explanatory variables as the explanatory variables were either dichotomous or categorical. Analysis of variance tests and a test of mean differences were used to examine the relationship between each outcome variable and the explanatory variables and to understand the patterns and differentials if any in each of the response variables (8 health sub-scales) and the selected respondents’ background and health characteristics. The F-test statistic and t-tests, where applicable were used to compare groups across the main study objectives. In addition, Kruskal Wallis test was computed for sub-scales that were less normally distributed, while the Levene’s test was used to examine equality of variance for the 8 health sub-scales. Eight separate multivariate linear regression models were fitted adopted to investigate interrelationships between the different factors in helping to explain each of the eight outcomes. Explanatory variables that were not significant in bivariate analyses were excluded from their respective model build up. Explanatory variables that had a significant relationship at the bivariate level but not significant after the initial fitting of the model were excluded and the model re-fitted. However, variables such as age, education and wealth status that are theoretically based and or found to be significant in other studies were retained in the final model whether its influence was significant or not. All the analyses were performed using Stata 12 SE [49].

## Results

Table 1 presents descriptive statistics of the eight SF-36 scales distribution of general health among the study sample. The health sub-scales were scored such that higher values represent better health, ranging from zero indicating poorer health, to 100, representing good health. The table shows that the distribution of the eight scales tended to be negatively skewed, with more respondents scoring among the more favourable health states. Skewness was more notable among scales defining role, social and physical limitations. The results show that on average respondents scored their health status lowest for vitality, followed by general health, bodily pain and mental health. Substantial ceiling effects were observed for role emotional (77.8%), role physical (71.5%), social functioning (58.8%) and physical functioning (46.7%). The table further shows the correlations between the SF-36 eight health sub-scales and their respective internal reliability test within each sub-scale. The inter-scale correlations revealed that each of the scale is positively but not highly correlated with each other (values < 0.60). The physical functioning sub-scale maintained a moderate association with General health, social functioning, vitality, role physical and bodily pain, while maintaining a weak correlation with mental health and role emotional sub-scales. On the other hand, mental health sub-scale maintained a moderate association with vitality and role emotional. Weak evaluation of the inter-scale correlations demonstrate that each scale measures a distinct concept of health. The internal reliability tests for each sub-scale using Cronbach alpha tests ranged between .69 for vitality and .94 for role physical. The internal consistency reliability estimates of ≥.65 for group comparison was achieved by all sub-scales. A split-half reliability test with different splits to compare the stability of the eight health sub-scales yielded 0.76 and 0.79 respectively, confirming the stability of the SF-36 instrument within the Ghanaian context [50].

**Table 1:** Number of items, means scores, rotated principal components associations between the eight SF-36 scales and among women in Accra, 2008

Scale	Transformed scores (0 - 100) for the eight SF-36 sub-scales							
	PF	RP	BP	GH	SF	VT	RE	MH
Items	10	4	2	5	2	4	3	5
Mean	85.2	78.3	72.9	71.8	87.7	70.7	82.2	77.4
Standard Deviation (SD)	21.0	37.9	23.9	19.7	20.4	15.3	35.8	14.8
Median	95.0	100.0	74.0	75.0	100.0	70.0	100.0	80.0
Skewness	−1.9	−1.4	−0.5	−0.6	−1.9	−0.7	−1.7	−0.8
% floor	0.9	15.8	0.2	0.0	0.6	0.0	14.0	0.0
% ceiling	46.7	71.5	25.7	5.8	58.8	1.9	77.8	5.5
	Inter scale correlation matrix and reliability coefficients (SF-36)							
Physical Functioning (PF)								
Role Physical (RP)	.51							
Bodily Pain (BP)	.46	.46						
General Health (GH)	.57	.43	.53					
Social Functioning (SF)	.54	.49	.46	.45				
Vitality (VT)	.52	.42	.56	.57	.46			
Role Emotional (RE)	.25	.40	.28	.27	.45	.33		
Mental Health (MH)	.26	.25	.34	.38	.37	.56	.41	
Cronbach alpha (α)	.93	.94	.73	.81	.76	.69	.93	.71
Split-half reliability test								
Split A	.76							
Split B	.79							
Observed cases (N)	2809							

A brief description of the explanatory variables is presented in Table 2. The sample distribution shows that more than half of the respondents were less than 40 years, while nearly 17% were aged 60 years and older. Seventeen percent of respondents had had no formal education, nearly 13% having had primary education, while 28% had had senior secondary or higher education. The results revealed that 18% of respondents had never been married, 54% currently married and 28% formerly married (separated, divorced or widowed). Twenty-one percent of women reported as nulliparous, while almost equal proportions (25%) had had 3-4 children or five or more children. Ethnic composition of the sample portrays Accra as a cosmopolitan area with an almost even spread of all ethnic groups represented. The study results showed nearly 28% of the sampled respondents were overweight, with an additional 36% being obese. Nearly 58% of respondents reported experiencing some form of disease symptoms four weeks prior to the survey. The results further showed that nearly one out of four respondents reported having poor social network relationships.

**Table 2:** Socio-demographic and health characteristics of respondents

Indicator	Percent	Number
Age groups		
20-29	24.5	687
30-39	26.4	742
40-49	19.6	552
50-59	12.9	362
60 and older	16.6	465
Marital status		
Currently married	54.2	1523
Formerly married	27.6	776
Never married	18.1	509
Parity		
None	20.7	582
1-2	29.4	828
3-4	25.4	714
5 or more	24.5	690
Ethnicity		
Ga-Dangme	38.5	1080
Akan	33.2	934
Ewe	14.1	395
Other ethnic groups	14.2	400
Educational Level		
None	17.1	481
Primary	12.8	360
Junior High School	42.0	1179
Secondary/higher	28.1	789
Wealth status		
Poorest	19.3	542
Poorer	21.9	614
Middle	20.9	587
Richer	20.5	576
Richest	17.4	490
Disease symptom		
No symptom	42.3	1187
Single	24.5	690
Multiple	33.2	932
BMI status		
Underweight	3.1	87
Healthy weight	31.7	891
Overweight	27.9	784
Obese	36.1	1014
Missing	1.2	33
Social network		
Good	76.1	2138
Poor	23.9	671
Total	100.0	2809

Table 3 presents the mean distribution of the eight health sub-scales by age, education and wealth status and their associated tests of significance are presented in Table 3. The summaries were statistically significant for all eight SF-36 sub-scales for age, education, parity, disease symptom and social networks at *p* < 0.001. Wealth status of the respondents did not maintain a significant relationship with Role Physical health sub-scale. Similarly, obesity status and ethnicity of the respondents did not maintain significant relationships with Bodily Pain and Role Emotional sub-scales. The results reveal that age is inversely related to health status, with very high values for the younger age groups (healthy scores) which decrease steadily in the older age groups. Each of the eight health sub-scales showed different relationships with age ranging from as high as 37.5 percentage points for physical functioning and as low as 3.0 percentage points variation between the oldest and youngest age groups for mental health scores. Overall PF, RP and GH sub-scales showed the widest variations across the various age groups. These variations were tested using the F-test statistic ratio of analysis of variance (one-way Anova) to discover whether there were any significant differences between the various mean scores for each of the health sub-scales and the various age groups. The observed distributions were further tested for differences in means using multiple comparisons mean differences test. The results indicate that overall, there were significant mean differences between the youngest age group (20 -29) and the oldest age group (60+) for all eight health sub-scales. For other age groupings however, there were some variations in their mean test results. Considering the PF health sub-scale assessment, there were significant differences between each pair of age groups apart from age groups 30 to 39 and 40 to 49 years. Similar patterns were observed for vitality and general health sub-scales. There were however, significant differences between each of the adjacent age groups compared, except for age groups 40-49 and 50-59 years. The pattern of association between role physical sub-scale and the various age groups showed that the test for mean differences for the younger age groups were not statistically significant (20-29, 30-39 and 40-49) but recorded significant mean differences compared to older age groups (50–59 and 60+). This means that role physical score decreases with age but this difference only becomes statistically significant as an individual moved beyond 49 years. There were no significant mean differences between age groups 30-39 and 40-49 years across all health sub-scales except for general health sub-scale.

**Table 3:** Analysis of variance and test of mean differences for eight health sub-scales by age, education and wealth status

Age group		Mean scores (standard deviation) for Short Form −36 Eight Health Sub-scales							
		PF	RP	BP	GH	SF	VT	RE	MH
20-29		96.4 (9.8)	86.9 (30.2)	79.0 (21.3)	79.4 (16.4)	92.1 (15.1)	76.3 (13.1)	86.2 (32.6)	79.1 (14.8)
30-39		93.1 (13.3)	86.6 (30.6)	76.2 (22.1)	75.7 (17.2)	89.4 (18.3)	73.0 (13.9)	82.8 (34.9)	77.3 (14.9)
40-49		89.6 (13.6)	80.1 (36.1)	72.3 (23.2)	70.7 (18.5)	89.3 (17.9)	70.6 (14.1)	79.7 (37.4)	77.0 (14.5)
50-59		78.2 (20.3)	72.3 (41.3)	70.8 (23.6)	68.1 (19.0)	87.2 (20.6)	67.3 (14.7)	82.4 (36.4)	76.9 (14.1)
60+		58.9 (28.6)	59.2 (46.0)	61.2 (26.5)	58.8 (22.1)	77.7 (27.9)	62.3 (17.4)	78.4 (38.9)	76.1 (15.8)
F-ratio		F[4, 2804] = 373.71*	F[4, 2808] = 47.38*	F[4, 2804] = 45.34*	F[4, 2808] = 97.46*	F[4, 2808] = 40.25*	F[4, 2808] = 71.14*	F[4, 2806] = 4.95*	F[4, 2808] = .3.24*
Multiple comparisons test of mean differences (MD) and standard errors (SE) between health sub-scales and each age group									
Broad age groups		MD [SE]	MD [SE]	MD [SE]	MD [SE]	MD [SE]	MD [SE]	MD [SE]	MD [SE]
20 -29	30 -39	3.27* [1.10]	1.84 [2.13]	3.11 [1.32]	3.86*[1.06]	3.17 [1.20]	3.45*[0.84]	4.75 [2.04]	1.89 [0.84]
	40 -49	6.24* [1.21]	5.73 [2.35]	6.24*[1.46]	8.43*[1.16]	2.68 [1.32]	5.36* [0.93]	7.07*[2.26]	2.07 [0.93]
	50 -59	17.73*[1.38]	14.24*[2.68]	8.26*[1.67]	11.52*[1.33]	4.82*[1.51]	9.04*[1.05]	4.99 [2.57]	2.78 [1.06]
	60+	39.98*[1.05]	29.71*[2.04]	18.86*[1.27]	21.87*[1.01]	16.52*[1.15]	14.78*[0.80]	9.78*[1.96]	3.50*[0.81]
30 -39	40 -49	2.97 [1.20]	3.89 [2.33]	3.13[1.45]	4.56*[1.16]	−0.48 [1.31]	1.91[0.92]	2.31[2.23]	0.17 [0.92]
	50 -59	14.46*[1.37]	12.40*[2.66]	5.14*[1.65]	7.65*[1.32]	1.66 [1.50]	5.59*[1.05]	0.23 [2.55]	0.88[1.05]
	60+	36.71*[1.04]	27.87*[2.02]	15.75*[1.25]	18.00*[1.00]	13.35*[1.14]	11.33*[0.79]	5.03 [1.94]	1.60[0.80]
40 -49	50 -59	11.49*[1.46]	8.51*[2.84]	2.01 [1.77]	3.09 [1.41]	2.14[1.60]	3.68*[1.12]	−2.08 [2.72]	0.71[1.12]
	60+	33.74*[1.16]	23.98*[2.25]	12.62*[1.40]	13.44*[1.12]	13.84*[1.27]	9.43*[0.89]	2.71[2.16]	1.43[0.89]
50 -59	60+	22.25*[1.34]	15.47*[2.59]	10.61*[1.61]	10.35*1.29]	11.70*[1.46]	5.74*[1.02]	4.79[2.49]	0.72[1.02]
Education		PF (SD)	RP (SD)	BP (SD)	GH (SD)	SF (SD)	VT (SD)	RE (SD)	MH (SD)
None		70.5 (28.8)	63.6 (44.6)	64.1 (25.9)	62.3 (21.6)	81.8 (24.8)	64.6 (16.7)	76.8 (39.8)	73.4 (14.8)
Primary		85.6 (21.0)	76.1 (39.1)	68.0 (25.0)	69.1 (20.2)	85.2 (22.1)	68.5 (16.1)	78.7 (38.8)	74.1 (15.8)
Junior High School		87.5 (18.8)	80.7 (35.9)	74.3 (22.5)	73.1 (18.7)	89.0 (18.9)	71.5 (14.2)	82.8 (35.4)	78.0 (14.1)
Secondary^+^		91.3 (16.6)	86.4 (31.3)	78.3 (22.4)	76.9 (17.6)	90.3 (18.1)	74.0 (14.4)	86.3 (31.9)	80.5 (16.7)
F-ratio		104.02 F[3, 2810]*	38.93 F[3, 2810]*	43.09 F[3, 2806]*	62.23 F[3, 2810]	21.99 F[3, 2810]	44.56 F[3, 2810]	9.15 F[3, 2808]	30.67 F[3, 2810]
Kruskal Wallis χ^2^		224.0* (3)	98.15*(3)	95.10*(3)	153.87*(3)	54.98*(3)	104.47*(3)	24.24*(3)	87.67*(3)
Multiple Comparisons method test of mean differences (MD) and standard errors (SE) between educational levels in each health sub-scale									
None	Primary	−17.92*[1.61]	−13.62*[2.64]	−5.48*[1.63]	−7.78*[1.34]	−6.34*[1.48]	− 5.46*[1.05]	− 2.23 [2.47]	−0.52[1.00]
	JHS	−21.44*[1.20]	−17.66*[1.98]	11.19*[1.22]	−12.28*[1.00]	−10.69*[1.11]	−8.79*[0.78]	−7.49*[1.85]	−.4.89*[0.75]
	Sec +	−25.38*[1.30]	−23.97*[2.13]	−15.60*[1.32]	−16.32*[1.08]	− 11.86*[1.20]	−11.16*[0.84]	− 10.09*[1.99]	− 7.34*[0.81]
Primary	JHS/	−3.52 [1.47]	−4.04 [2.41]	−5.70*[1.49]	− 4.71*[1.22]	− 4.35*[1.36]	− 3.32*[0.96]	−5.26[2.25]	− 4.38*[0.92]
	Sec +	−7.46*[1.54]	−10.35*[2.54]	− 10.11*[1.57]	− 8.74*[1.29]	− 5.52*[1.43]	− 5.69*[1.01]	−7.87*[2.37]	− 6.82*[0.96]
JHS	Sec +	−3.94*[1.12]	−6.31*[1.84]	−4.41*[1.13]	− 4.03*[0.93]	−1.17 [1.03]	2.37*[0.73]	−2.60 [1.72]	− 2.44*[0.70]
Wealth status		PF (SD)	RP (SD)	BP (SD)	GH (SD)	SF (SD)	VT (SD)	RE (SD)	MH (SD)
Poorest		81.7 (24.2)	75.0 (39.4)	70.4 (24.7)	67.1 (21.0)	84.7 (22.6)	67.6 (16.2)	77.3 (39.3)	73.4 (15.2)
Poorer		86.2 (20.4)	78.3 (37.3)	71.1 (23.7)	70.3 (19.9)	87.7 (19.3)	69.1 (15.2)	81.2 (36.3)	75.4 (14.9)
Middle		86.5 (20.7)	79.2 (37.3)	73.4 (23.9)	73.0 (19.0)	87.9 (20.1)	71.4 (13.8)	81.6 (36.6)	77.4 (14.8)
Richer		86.2 (21.8)	79.4 (37.7)	73.3 (23.8)	73.4 (18.9)	89.1 (19.8)	72.9 (14.7)	84.1 (34.8)	79.8 (14.2)
Richest		85.3 (21.8)	79.9 (37.4)	76.7 (23.0)	75.6 (18.8)	89.1 (19.7)	72.6 (15.4)	86.6 (31.5)	81.0 (14.3)
F ratio		F(4, 2809) = 4.63*	F[4, 2809] = 1.47	F[4, 2807] = 5.48*	F[4, 2809] = 15.23*	F[4, 2809] = 4.31*	F[4, 2809] = 12.64*	F[3, 2808] =4.83*	F[3, 2810] =24.18*
Multiple Comparisons test of mean differences (MD) and standard errors (SE) between each pair of SES in each health sub-scale									
Poorest	Poorer	−4.47*[1.28]	N/A	−0.69 [1.41]	−3.26*[1.15]	−2.96 [1.20]	−1.41 [0.89]	− 3.80 [2.11]	− 2.04 [0.86]
	Middle	−4.82*[1.29]		−2.93 [1.42]	−5.94*[1.16]	−3.24 [1.21]	−3.75*[0.90]	− 4.26 [2.14]	− 3.97*[0.87]
	Richer	−4.45*[1.30]		−2.82 [2.43]	−6.37*[1.17]	−4.42*[1.21]	−5.22*[0.90]	−6.75* [2.15]	− 6.44*[0.88]
	Richest	−3.59 [1.35]		−6.25*[1.49]	−8.59*[1.22]	−4.44* [1.27]	− 4.94*[0.94]	−9.19* [2.24]	− 7.61*[0.91]
Poorer	Middle	−0.35 [1.25]		−2.25 [1.38]	−2.69 [1.13]	−0.28 [1.17]	− 2.35 [0.87]	− 0.46 [2.07]	−1.92 [0.85]
	Richer	0.02 [1.26]		− 2.13 [1.38]	−3.11* [1.13]	−1.46 [1.18]	−3.81*[0.87]	− 2.95 [2.08]	−4.40*[0.85]
	Richest	0.88 [1.32]		−5.56* [1.44]	−5.34*[1.18]	−1.48 [1.23	−3.54* [0.91]	− 5.39 [2.17]	−5.56*[0.89]
Middle	Richer	0.37 [1.27]		0.12 [1.40]	−0.42 [1.22]	−1.18 [1.19]	−1.47 [0.88]	−2.49 [2.10]	−2.47* [0.86]
	Richest	1.23 [1.33]		−3.32 [1.47]	−2.65 [1.19]	−1.20 [1.24]	−1.19 [0.92]	−4.93 [2.20]	−3.64*[0.90]
Richer	Richest	0.86 [1.34]		−3.43 [1.47]	−2.23 [1.20]	−0.02 [1.25]	0.28 [0.93]	−2.44 [2.21]	−1.17 [0.91]

Note: Testing for significant mean differences between individual pairs of group means. *SD* Standard deviation, *N/A* Not applicable; *p < 0.05

*PF* Physical Functioning, *RP* Role Physical, *BP* Bodily Pain, *GH* General Health, *SF* Social Functioning, *RE* Role Emotional, *MH* Mental Health

Table 3 further show significant differences between each of the eight health sub-scales and education among the study population. The variation in mean differences between women with secondary or higher education compared to those with no formal education ranged between 22.8 and 7.1 percentage points for the role emotional and mental health sub-scales respectively. The multiple tests of mean differences the categories of education on the eight-health sub-scales, revealed that each category of educational level (no education, primary, JHS and secondary or higher) were significantly different from each other at *p* < 0.05 for bodily pain, general health and vitality sub-scales. Physical functioning and role physical sub-scales revealed significant mean differences between all pairs of educational categories, except for between primary and Junior high school, which could not be statistically substantiated at *p* < 0.05. Similarly, for role emotional sub-scale, the mean differences between respondents with no education versus primary level and those with JHS/middle compared against those with secondary or higher level could not be statistically differentiated at *p* < 0.05. The educational group variations showed that among respondents with no formal education, a higher proportion scored their health lowest on GH, RP, BP and VT.

The results of the associations between each of the eight health sub-scales and wealth status of the individual reveals that average score for the health sub-scales did not vary much by the socio-economic status of the respondents. The percentage variation ranged from as low as 3.6 to 9.3 percentage points for physical functioning and role emotional sub-scales respectively between the poorest and the richest groups. The anova test results between role physical and wealth status was not significant, hence, role physical sub-scale was excluded from the multiple comparison tests of mean differences. The results of the mean differences show that wealth status was positively related to the remaining seven health sub-scales. The mean difference between the richer and richest wealth quintiles was insignificant across all health sub-scales. The mean comparison tests show that within mental health sub-scale, each pair of the adjacent mean scores were significantly different from each other, apart from the difference between ‘middle’ and ‘richer’ quintiles. The test of mean differences for general health sub-scale showed that, the mean value for women of the ‘poorest’ group were significantly different from the mean scores obtained by the ‘poor’, ‘middle’, ‘richer’ and ‘richest’ groups at *p* < 0.05, as well as the mean values for the ‘poor’ against the ‘richer’ and the ‘richest’ groups. Vitality health sub-scale registered five significant mean differences between ‘poorest’ and ‘middle’; ‘poorest’ and ‘richer’, ‘poorest and richest’ groups as well as between ‘poor’ versus ‘richer’ and ‘richest’ groups at *p* < 0.05.

Multivariate linear regression models were fitted for each of the eight SF-36 health sub-scales to further explore the significance of the predictors. The factor variables included in the investigation were broad age group categories, educational levels, number of children ever born, socio-economic status, ethnicity, BMI status, disease symptoms experienced and social networking. The estimated coefficients, standard errors and 95% significance level estimates are presented in Table 4. Variance inflation factor (VIF) score which measures the presence of collinearity were computed for the independent variables for each of the eight models. The VIF for each of the predictor variables yielded an average score of ≤2.87 and a corresponding tolerance level of less than 0.55. These indicate that multicollinearity issues were not problematic among the various explanatory variables. Controlling for all variables, the results revealed that the combined effect of the predictor variables helped to explain between 10 and 40% of the variations in the each of the eight sub-scales. Was explained by a portion of variance ranging from 10.0% for role emotional sub-scale to 39.5% for physical functioning. There appears that physical functioning is the most important SF-36 sub-scale significantly influenced by the set of predictor variables explaining about 41%.

**Table 4:** Estimated beta coefficient, standard errors and 95 level of significance for eight health sub-scales among women in Accra, 2008

Indicators	PF	RP	BP	GH	SF	VT	RE	MH
	β (SE)	β (SE)	β (SE)	β (SE)	β (SE)	β (SE)	β (SE)	β (SE)
Constant	93.60(1.5)	87.39(3.0)	84.21(1.7)	77.94(1.6)	93.84(1.6)	78.17(1.2)	89.99(2.8)	78.07(1.2)
Age group *(20-29 omitted)*								
30 -39	−2.55(0.8)	−0.14(2.0)	−1.36(1.2)	−3.15(0.9)	− 2.84(1.0)	− 2.77(0.8)	− 3.84(2.0)	n/s
40 -49	−4.28(1.0)	− 2.57(2.3)	− 2.90(1.4)	−6.74(1.0)	−2.48(1.2)	− 3.66(0.9)	−5.13(2.3)	n/s
50 -59	− 14.54(1.4)	−10.28(2.8)	− 4.26(1.6)	−9.47(1.3)	− 4.37(1.4)	−7.11(1.1)	− 3.02(2.6)	n/s
60+	− 30.95(1.3)	−19.47(2.6)	− 11.95(1.6)	− 16.19(1.6)	−11.06(1.4)	− 10.22(1.0)	− 4.46(2.6)	n/s
Education *(no education omitted)*								
Primary	4.35(1.2)	5.19(2.9)	−0.96(1.5)	1.67(1.3)	−0.06(1.3)	0.45(1.0)	0.90(2.5)	−0.23(1.0)
JHS	4.41(1.0)	6.58(2.7)	3.70(1.3)	3.40(1.1)	2.46(1.1)	1.69(0.8)	3.06(2.1)	2.69(0.9)
Secondary or higher	5.50(1.3)	9.82(2.7)	5.43(1.5)	4.21(1.2)	3.01(1.3)	2.33(1.0)	4.90(2.4)	3.80(1.0)
Ethnicity *(Ga-Dangme omitted)*								
Akan	2.11(0.8)	n/s	n/a	3.30(1.0)	−0.82(0.8) ǂ	−0.79(0.6)	n/a	n/s
Ewe	2.03(1.0)	n/s	n/a	2.60(1.4)	0.13(1.1) ǂ	−1.86(0.8)	n/a	n/s
Other	0.45(1.0)	n/s	n/a	1.76(1.7)	0.80(1.1) ǂ	−1.48(0.8)	n/a	n/s
Wealth status *(Poorest omitted)*								
Poorer	1.11(0.9)	n/a	−1.59(1.4) ǂ	1.09(1.2)	1.32(1.1)	0.06(0.8)	2.71(2.0) ǂ	1.27(0.8)
Middle	0.97(0.9)	n/a	−1.19(1.5) ǂ	2.54(1.2)	0.90(1.1)	1.53(0.8)	1.31(2.1) ǂ	2.22(0.9)
Richer	0.17(1.2)	n/a	−2.14(1.5) ǂ	2.37(1.4)	1.46(1.1)	2.46(0.9)	3.13(2.1) ǂ	4.22(0.9)
Richest	0.52(1.1)	n/a	0.25(1.5) ǂ	4.28(1.5)	1.71(1.2)	2.16(0.9)	3.69(2.3) ǂ	4.39(1.0)
Disease symptom *(no symptom omitted)*								
Single	−3.61(0.8)	−4.54(1.6)	−8.56(1.0)	−4.24(1.1)	−1.33(0.9)	−3.67(0.7)	−4.98(1.7)	−2.30(0.7)
Multiple	−9.57(0.9)	−20.54(1.8)	−19.82(1.1)	−10.36(1.2)	− 8.19(1.0)	−9.25(0.5)	− 18.58(1.5)	− 8.60(0.6)
BMI status *(healthy-weight omitted)*								
Underweight	−2.66(2.1)	n/s	n/a	n/s	n/s	n/s	n/a	n/s
Overweight	−0.83(0.8)	n/s	n/a	n/s	n/s	n/s	n/a	n/s
Obese	−4.09(0.7)	n/s	n/a	n/s	n/s	n/s	n/a	n/s
Social network *(good omitted)*								
Poor	−4.15(0.8)	−12.57(2.1)	−8.77(1.2)	−8.91(1.0)	−14.3(1.2)	−6.00(0.6)	− 14.77(1.5)	−5.24(0.6)
Adjusted R^2^	40.7%	16.0%	24.3%	25.1%	19.2%	22.1%	10.4%	14.1%
N	2758	2808	2805	2808	2809	2808	2807	2809

*Abbreviations*: *JHS* Junior High School; *Note*: *n/s* Not significant at p < 0.05, *Omitted* Reference category, *n/a* Not application. Details of the significance are shown in Table 5

ǂ Significant at p < 0.1

The results show that controlling for all other factors education and disease symptom experienced had the greatest influence on all eight health dimensions, while BMI status had the least influence on the eight SF-36 health sub-scales. With BMI retaining a statistically significant relationship with only the physical functioning sub-scale. Age maintained a statistically significant relation with seven of the eight sub-scales except for the mental health dimension. Wealth status and ethnicity retained significant relationship with three of the eight sub-scales. Accounting for all other factors, wealth status had a significant relationship with general health, vitality and mental health sub-scales. Ethnicity of the respondent maintained a statistically significant influence with general health, physical functioning and vitality sub-scales. Education and disease symptom experienced had pronounced influences on all eight health sub-scales.

Controlling for all other factors, there were significant differences among the eight health sub-scales between the various levels of education. Respondents with higher education reported significantly higher scores in all eight health dimensions. There were significant increases in good health status for respondents with higher educational level for Role Physical (*p* < 0.001) and the lowest mean increase for vitality health sub-scale (*p* < 0.001).

Disease symptom experienced by respondents also maintained a significant net effect on all the eight health sub-scales. Respondents who experienced multiple disease symptoms reported worse health decreasing the each of the eight health sub-scale between 8.6 through 20.6 percentage points compared with the mean of those with no disease symptom. The greatest decrease was observed for role physical (20.5), closely followed by bodily pain but lowest on social functioning (see Table 4). There were also significant differences between respondents who experienced a single disease symptom and those who did not, with the former reporting a decrease in health status on all the eight sub-scales.

Age of respondents maintained a significant net effect with seven health sub-scales, except mental health status. Age of the respondent maintained a different impact with the sub-scales. As an individual progressed beyond 49 years, there was a steady decrease on all the health sub-scales, with the highest decline in physical functioning and lowest in the mental health dimensions. The results further show that comparing the influence of age groups 30-39 and 40-49 years to the reference group (20-29 years) independently, on all the eight sub-scales, they rated their health status worse off, but was not statistically significant for physical functioning. Respondents of 40-49 years maintained a different impact, reporting a much lower decrease on social functioning, role emotional and mental health than respondents of 30-39 years, although they reported worse health status than the reference. However, beyond this age increase in age was associated with a significant decrease in health status than the preceding age group as expected.

Table 5 summarises the net effect and direction of the contribution of the explanatory variables on all eight health sub-scales. Education maintained positive net significant effect with all health sub-scales. Social network maintained significant net effect relationship with four sub-scales. This include social functioning, vitality, role emotional and mental health. Respondents who maintained poor social networking reported significantly low health on role emotional, closely followed by social functioning and highest on mental health sub-scales holding all predictor variables to their baseline values. Wealth status and ethnicity maintained significant relationship with three of the eight SF-36 sub-scales. Wealth status had a significant positive relationship with general health, vitality and mental health sub-scales. The higher the wealth status of the individual the higher or better assessment of their health status on those three scales. Ethnicity of the respondent maintained a statistically significant influence with general health, physical functioning and vitality sub-scales but in different directions. Sampled respondents of the Akan, Ewe and other ethnic groups rated their health status better than women of Ga-Dangme ethnic group. However, women of all other ethnic groupings other than Ga-Dangme (the reference group) rated their health status lower on vitality sub-scale.

**Table 5:**
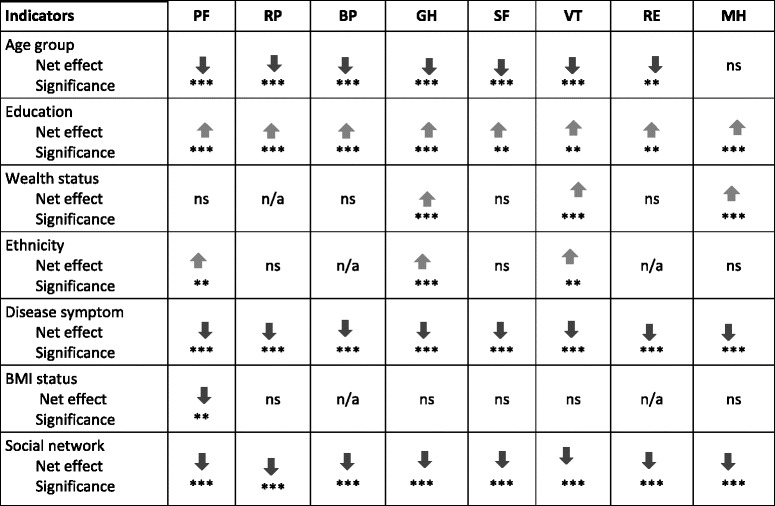
Influence of demographic, socio-economic and health-related factors on SF-36 sub-scales: multivariate analyses results

Variable magnitude of significance *** *p* < 0.01, **p < 0.05; *n/a* Not applicable, *ns* Not significant

Abbreviations: *PF* Physical Functioning, *RP* Role Physical, *BP* Bodily Pain, *GH* General Health

*SF* Social Functioning, *VT* Vitality, *RE* Role Emotional, *MH* General Mental Health

## Discussion

The paper set out to assess the intra-urban health differentials and possible determinants that might exist among women 20 years or older living in the city of Accra, by evaluating the association between eight health sub-scales obtained from SF-36 and selected demographic, socio-economic and health-related variables. The results from the descriptive, bivariate and multivariate regression models revealed different impacts on the various health dimensions.

The results revealed varied levels of health status amongst the women, measured by age, educational level, wealth status, ethnicity, disease symptom experienced and social networks, maintained. The results showed that on average respondents scored lowest on vitality or fatigue, bodily pain and general health issues. Women in times past were mainly home-makers and taking care of their young ones. In recent times, the changing economic situation have seen a lot of these women taking up jobs outside the home, acting as heads of households and as bread winners. They still combine these new chores with their socially assigned roles of can be daunting, very stressful and have a toll on their wellbeing and general health status. The observed high overweight and obesity rates among the sampled population is alarming. Overweight and obesity are risk factors for hypertension, type II diabetes, cardiovascular diseases and other chronic conditions which have a strain on individual health status. These observed rates may trigger other health conditions among the study population and invariably affect their physical functioning among others. Surprisingly obesity status contrary to expectation did not have any significant influence on bodily pain, role physical nor emotional.

Simultaneous multiple regression analyses confirmed disease symptom, education and age as independent factors having a great impact on almost all the eight health sub-scales. Controlling for all variables, the results revealed that each of the eight health sub-scales was explained by variance explained by the adjusted regression models ranged between 10.1% for role emotional and 40.7% for the physical functioning. It was lowest on role emotional and highest on physical functioning health scales.

Age was relatively more important factor affecting physical functioning, vitality and general health than mental health and role emotional. The effect of age was consistent with expectation that health deteriorates with increasing age [51, 52]. This was expected, as people progress the age ladder, they are less likely to be full of energy, climbing flights of stairs without limitations, and undertaking vigorous or strenuous activities than the younger age groups. The strong negative relationships between increasing age and poor physical health statuses can be explained as old age impacts more on physical functioning and roles that one can easily perform. Again, as individuals’ progress in years they are more susceptible to live with disabilities.

The study results further revealed fewer variations amongst the various age groups in relation to mental health and role emotional sub-scales; when compared to the other scales. This could possibly be that, older women have wider networks, family support and are more stable emotionally or maintain a positive attitude towards life in general than the younger women. Women in the younger age groups are probably nursing mothers, who grapple with childcare, social roles and responsibilities, as well as make a living especially in female-headed households and are more likely to experience stress and poor mental health. Thus, low mental health status is age irrelevant compared to the other sub-scales.

Education serves as an important mechanism for enhancing the health status of individuals as educated women tend to have better understanding of their health needs and have a greater ability to seek medical attention on time and on a regular basis. Women with higher education also tend to secure more reliable jobs with higher income than, women with no education. The results from the analyses indicated that health patterns and differentials across the eight sub-scales all support the fact that education is a critical factor in assessing women’s health status. Women with no education or lower levels of education turn to be less self-sufficient, not able to take independent decisions regarding their health need and hence reporting worse health on all eight sub-scales. Higher education is instrumental in achieving and maintaining good health status among the study population.

Women who experienced multiple disease symptoms had lower scores on all eight sub-scales. Experiencing multiple disease symptoms is likely to have adverse effect on physical functioning, bodily pain, roles that one can perform and general health status. Such respondents without seeking health care or receiving treatment are more likely to develop a chronic condition, be recurrent and may eventually lead to increased functional and emotional disability.

The results also show a clear socio-economic status gradient existed for almost all sub-scales except role physical. Women who scored poor health increased for women from poorer households and this finding agrees with those of [53]. However, at multivariate analysis when other variables were included in the model, wealth status showed significant associations with only mental health, general health and vitality sub-scales. This suggests that being on a higher socio-economic status enables one to enjoy better emotional well-being but not physical functioning.

It is worthwhile noting some of the limitations of the study. The study is focussed on women alone, hence one cannot make comparison of gender perspective of self-assessed health among adult residents in the city of Accra. Although, the study focused on women, they serve as primary caregivers to their families thus lessons learned from their perceptions would help address some of the health needs among the population. Although, the study draws on the second wave of the Women’s Health Study of Accra, this paper is based on the cross-sectional study thus causality regarding intra-urban health issues cannot be inferred. In addition, the two-data points do not permit a longitudinal study but provides useful way of exploring self-assessed health among the study population. Noting these limitations, the study findings help to understand and appreciate differentials in health and wellbeing among the study population from socio-demographic and disease symptom perspective. Without being in the clinical setting and not having complete and accurate data using the SF-36 eight sub-scales helps to assess the current health status any study population. Findings help to appreciate that health is multifaceted and are influenced differently by the same strenuous factors, hence people living in the same geographic region need not experience health and wellbeing the same way.

## Conclusion

This study has demonstrated using socio-demographic characteristics to describe and gauge how respondents in the general population assess their health status. A limitation of the study worth noting is that it is based on cross-sectional data. Therefore, a direct causal relationship cannot be inferred between the explanatory variables and various health status measures. However, the analysis presented gives evidence of the relationship between demographic, socio-economic and health-related indicators and general health outcomes assessed using eight SF-36 health sub-scales. The findings from these analyses have shown that health issues are not restricted to health practitioners and health policy makers alone but they are multi-faceted requiring socio-cultural and economic policy intervention as well. Investing in women’s education to higher levels is important to improve overall health status. Ageing has an increasing relationship with lower self-health assessment and this group should be given the needed attention and support. There is the need for systematic and useful intra-urban health statistics to help assess progress over time, as the effect of the significant explanatory factors influencing health status maintained different pathways. There is also the need for more effective collaboration across various government institutions and agencies including ministry of health, education, women and children affairs, economic and employment and other important stakeholders to improve the health and well-being of women in general.
